# Short Bowel Patients Treated for Two Years with Glucagon-Like Peptide 2 (GLP-2): Compliance, Safety, and Effects on Quality of Life

**DOI:** 10.1155/2009/425759

**Published:** 2009-07-05

**Authors:** P. B. Jeppesen, P. Lund, I. B. Gottschalck, H. B. Nielsen, J. J. Holst, J. Mortensen, S. S. Poulsen, B. Quistorff, P. B. Mortensen

**Affiliations:** ^1^Department of Gastroenterology, Copenhagen University Hospital (Rigshospitalet), 2121 Copenhagen, Denmark; ^2^Department of Anesthesia, Copenhagen University Hospital (Rigshospitalet), 2100 Copenhagen, Denmark; ^3^Department of Biomedical Sciences, The Panum Institute, 2200 Copenhagen, Denmark; ^4^Department of Clinical Physiology and Nuclear Medicine, Copenhagen University Hospital (Rigshospitalet), 2100 Copenhagen, Denmark

## Abstract

*Background and aims*. Glucagon-like peptide 2 (GLP-2) has been shown to improve intestinal absorption in short bowel syndrome (SBS) patients in a short-term study. This study describes safety, compliance, and changes in quality of life in 11 SBS patients at baseline, week 13, 26, and 52 during two years of subcutaneous GLP-2 treatment, 400 microgram TID, intermitted by an 8-week washout period. *Methods*. Safety and compliance was evaluated during the admissions. The Sickness Impact Profile (SIP), Short Form 36 (SF 36), and Inflammatory Bowel Disease Questionnaire (IBDQ) evaluated quality of life. *Results*. The predominant adverse event was transient abdominal discomfort in 5 of 11 patients, but in 2, both suffering from Crohns disease, it progressed to abdominal pain and led to discontinuation of GLP-2 treatment. One had a fibrostenotic lesion electively resected at the jejuno-ascendo-anastomosis. The investigator excluded a patient due to unreliable feedback. Stoma nipple enlargement was seen in all 9 jejunostomy patients. Reported GLP-2 compliance was excellent (>93%). GLP-2 improved the overall quality of life VAS-score (4.1 ± 2.8 cm versus 6.0 ± 2.4 cm, *P* < .01), the overall SIP score (10.3 ± 8.9% versus 6.2 ± 9.5%, *P* < .001), the mental component of the SF-36 (45 ± 13% versus 53 ± 11%, *P* < .05), and the overall IBDQ score (5.1 ± 0.9 versus 5.4 ± 0.9, *P* < .007) in the 8 patients completing the study. *Conclusions*. Long-term treatment with GLP-2 is feasible in SBS patients, although caution must be exercised in patients with a history of abdominal pain. Although conclusions cannot be made in a noncontrolled trial, the high reported compliance might reflect a high treatment satisfaction, where the clinical benefits of GLP-2 may outweigh the discomforts of injections.

## 1. Introduction

Until recently, the medical treatment options in short bowel patients with intestinal insufficiency or failure were based on antidiarrhoeals (codeine, opium, and loperamide) and anti-secretory agents (H_2_-receptor blockers and proton pump inhibitors). Now, growth factors such as growth hormone and glucagons-like peptide 2 have been suggested as treatment options in these patients. In fact, the FDA approved growth hormone (Zorbtive) for the treatment of short bowel syndrome (SBS) patients with intestinal failure in 2003. The effects of growth hormone on intestinal function are controversial, and serious side effects, due to the systemic effects of growth homone, such as myalgia, arthralgia, gynecomastia, carpal tunnel syndrome, nightmares, and insomnia, are reported even in the relative short-term studies [1]. These side effects may limit the acceptance of long-term administration and may impair the quality of life of the HPN patients. The more targeted effects of Glucagon-like peptide 2 (GLP-2) and a dipeptidyl-peptidase IV (DPP-IV) degradation-resistant gly-2 GLP-2 analogue [2], Teduglutide, on intestinal function, and the relative few side effects described in short-term studies, seemingly make these agents more attractive [3, 4]. However, since they have only been used for maximum 35 days, the current study seeks to evaluate the feasibility and acceptance of long-term GLP-2 treatment in patients with intestinal insufficiency or failure. In this respect, compliance and safety issues were addressed in eleven patients, who were offered GLP-2 for subcutaneous injection TID for two consecutive years intermitted by an 8-week washout period. The concomitant physiological effects derived from this open-label, long-term GLP-2 study are presented separately. The current study also describes the longitudinal changes in quality of life evaluated by the Sickness Impact Profile (SIP) [5], the Short Form 36 (SF 36) [6], and the Inflammatory Bowel Disease Questionnaire (IBDQ) [7], and treatment satisfaction was measured.

## 2. Material and Methods

### 2.1. Patients

Eleven SBS patients (3 female, 8 male; 47 ± 11 years; remnant small bowel 157 ± 66 cm; 2 with a colon; 7 had intestinal failure, 3 receiving parenteral fluids and electrolytes exclusively and 4 receiving parenteral nutrition; 4 had intestinal insufficiency and did not need parenteral nutrition or fluid) were recruited to the study based on a fecal energy excretion exceeding 2.0 MJ/day (measured at a previous admission) or a remnant small bowel of 200 cm or less (measured preoperative from the ligament of Treitz). The short bowel syndrome was a consequence of inflammatory bowel disease in all patients. The patients did not have activity in their IBD as evaluated clinically, and none of the patients received maintenance medication. The individual patient characteristics at baseline are presented in Table 1.

### 2.2. Study Protocol

Over the two years, the patients were admitted at least eight times to the hospital for 72-hour evaluations. After the baseline evaluations, the patients were given native GLP-2, 400 mcg TID, subcutaneous, for one year. For these studies we employed synthetic human GLP-2, corresponding to human proglucagon 126–158, custom-synthesized by PolyPeptide Laboratories GmbH, Wolfenbüttel, Germany, as described previously [3]. The patients were scheduled for readmissions at 13, 26, and 52 weeks (abbreviated Y1-W13, Y1-W26, Y1-W52, resp.). After the first readmission at week 13, the patients were given an option to test a double dose of GLP-2, 800 mcg TID for 3 weeks. The patients, who accepted, were readmitted for an extra 72-hour nutrient balance study at week 17 (Y1-W17). After completing this evaluation, the original GLP-2 dose was reintroduced. GLP-2 treatment was discontinued for 8 weeks after the first 52 weeks of treatment. After a 72-hour follow-up evaluation, the GLP-2 treatment, 400 mcg TID, was re-introduced and evaluations were repeated during admission at 13, 26, and 52 weeks during the second year of treatment (abbreviated Y2-W13, Y2-W26, Y2-W52, resp.). In relation to the week 26 readmission, during the second year of GLP-2 treatment, the patients were given cholylsarcosine bile acid replacement therapy, 2 g TID, two days prior to the admission and during the 72-hour balance studies. Cholylsarcosine was introduced since it has been described to increase fat absorption in SBS patients. Cholylsarcosine was packed into gelatine capsules (250 mg/capsule) as described previously [8]. Cholylsarcosine was taken in relation to the three main meals and in conjunction with subcutaneous GLP-2 injections.

#### 2.2.1. Compliance and Safety

The patients were asked to fill in deviations from their daily medical and parenteral prescriptions in a patient diary, and GLP-2 compliance was evaluated and crosschecked by counting returned empty vials. The patients also described effects and side effects of GLP-2 treatment in this diary. During the 72-hour admissions, the patients were monitored for safety (adverse events, physical exams, injection site examinations, and standard laboratory results).

#### 2.2.2. Quality of Life and Treatment Satisfaction

During admissions, the patients were instructed to fill in three validated questionnaires regarding quality of life: The Sickness Impact Profile (SIP) [5], the Short Form 36 (SF 36) [6], and the Inflammatory Bowel Disease Questionnaire (IBDQ) [7]. The SIP and SF 36 questionnaires are designed to be broadly applicable across types and severities of illness and across demographic and cultural subgroups. They were chosen for this study to provide a measure on the non-disease-specific function of the patients. The IBDQ was developed to measure subjective health status for patients with inflammatory bowel disease. This questionnaire is disease-specific and was chosen to focus on bowel-related symptoms and the impact on quality of life.

The SIP measures sickness-related dysfunction and is designed to cover patient perception of performance in areas of activity in everyday life. It contains 136 items in two main dimensions and 5 independent categories in areas of activity; Physical (ambulation and mobility, body care and movement), psychosocial (social interaction, alertness and emotional behavior, communication), and independent categories (sleep and rest, eating, work, home keeping, recreation, and pastimes). Patients were asked to endorse or check those statements that were in accordance with their present situation. No positive answers were equivalent to no behavioral dysfunction. The SIP percent scores of the dimensions and categories were obtained by summing the number of positive statements to the items in each dimension and category, dividing that sum by the total sum of the possible values and multiplying the quotient by 100. Zero percent indicates the best possible function (absence of dysfunction), whereas 100% indicates presence of all possible dysfunctional behavior. At the end of the SIP questionnaire patients were asked to mark their overall quality of life on a 9 cm visual analogue scale (VAS). At the left at 0 cm a miserable quality of life was indicated, whereas an ideal quality of life was indicated at 9 cm at the right end of the scale.

The SF 36 measures eight multi-item variables: physical functioning (10 items), role limitations due to physical problems (four items), pain (two items), general perception of health (five items), social functioning (two items), role limitations due to emotional problems (three items), mental health (five items), and energy and vitality (four items). For each variable items, scores are coded summed, and transformed on to a scale from 0 (worst possible health state measured by the questionnaire) to 100 (best possible health state). The physical component summary includes the four first items and the mental component summary includes the last four items.

The 32-item IBDQ questionnaire examines four aspects of patients' lives: symptoms directly related to the primary bowel disturbance (10 questions), systemic symptoms (5 questions), emotional (12 questions), and social function (5 questions). The response options for each question were framed as a seven-point scale at which 7 represented best function and 1 represented worst function. The scores of each aspect have been given as mean on the 7-point scale.

In addition to the three validated questionnaires regarding quality of life, an evaluation-form containing questions regarding treatment satisfaction in relation to GLP-2 treatment was given to the patients after the two years of GLP-2 treatment. At week 26 during the second year of GLP-2 treatment, where the patients also received oral bile acid replacement therapy, the patients were questioned weather they preferred subcutaneous GLP-2 (400 *μ*g TID) or oral capsules with cholylsarcosine (2 g ~ 8 capsules TID) on the assumption, that the effect was the same.

## 3. Ethics

The Ethics Committee for Medical Research in Copenhagen, Denmark, (KF 01-235/98) approved the protocol. Procedures followed were in accordance with the ethical standards of the Helsinki Declaration of 1975, as revised in 1983. Patients signed informed consent before entrance to the study.

## 4. Statistics

The differences between admissions periods were tested with a Friedman repeated measures analysis of variance on ranks on using the SigmaStat for Windows Version 2.0 (Copyright® 1992–1995, Jandel Corporation, Erkrath, Germany) in patients completing the study. For comparisons of admission periods to the baseline period, the Dunnett method was used as the post hoc test. The frequencies of confirmatory answers in the SIP questionnaire were compared by the chi-square, alternatively Fisher exact test. A value of *P* < .05 was considered significant.

## 5. Results

### 5.1. Compliance and Safety

The compliance reflects the tradeoff between the clinical benefits of receiving GLP-2 or possible secondary benefits perceived by the patient in relation to participating in the study versus the side effects, discomforts (from injections, frequent admissions, study-related invasive procedures, etc.), and potential risks in relation to treatment. In this respect 3 of the 11 patients did not complete the study.

Patient O. B. chose to discontinue GLP-2 after 232 days of treatment due to aggravation of chronic, intermittent, abdominal pain and nausea. A barium follow-through did not reveal a small bowel obstruction, and the patient had no clinical or biochemical evidence of activity in his Crohn's disease. The condition improved in relation to discontinuation of treatment. The patient had a compliance of 93% of injections prior to discontinuing GLP-2 treatment. 

Patient J. P. discontinued GLP-2 treatment at day 160 during the second year of the study due to signs of bowel obstruction. A barium follow-through revealed a short-segment relative obstruction at the jejuno-ascendo-anastomosis. An elective resection of a fibrostenotic lesion of less than 5 cm at the anastomosis was performed. There were no macroscopic or microscopic signs of active Crohn's disease. Compliance was 100% the first year and 94% until treatment was discontinued the second year. The patient did not want to continue GLP-2 treatment after the resection. 

Patient E. F. P. was excluded from the study by the investigator after 174 days of GLP-2 treatment during the second year of the study. The patient could not account for the dispensed GLP-2 vials and admitted to disposing them instead of returning them. At this point, it became evident that she had stopped filling in the patient diary, and that her reliability could be questioned due to an escalating abuse of analgesics. As evaluated by the returned vials and the diary, the compliance was 100% the first year of treatment. According to the patient, she had taken “most injections” during the second year of treatment until exclusion. She discontented with the decision to exclude her, since she had experienced positive effects of GLP-2.

In the remaining eight patients, who completed the study, the compliance was more than 94% during both years of GLP-2 treatment.

### 5.2. Adverse Events

Two study-related serious adverse events occurred. Patient G. L. experienced a distal bowel perforation following study-related biopsies taken through the ileostomy, prior to the initiation of GLP-2 treatment. A new ileostomy was created after resection of 5 cm of small bowel. After time for recovery from surgery, the patient insisted on inclusion in the study, but he refused the biopsy scheduled for week 52. Patient J. H. J. experienced prolonged venous bleeding from the stoma after having biopsies taken at week 52. In contrary to instructions, the patient had taken a GLP-2 injection just prior to having the biopsies taken. The bleeding stopped spontaneously, but the patient was given two transfusions due to a drop in hemoglobin from 7.2 mmol/L to 5.3 mmol/L. 

Other adverse events included a transient tender abdomen in relation to the initiation of GLP-2 treatment described in 5 of 11 patients (Patients H. R. M., O. B., J. P., E. F. P., J. E., and U. D. J.). Reduced appetite and peripheral edema was described in 2 of 11 patients (Patients H. R. M. and E. F. P.). Six out of 11 patient reported, that they urinated more than habitually in relation to GLP-2 treatment (Patients H. R. M., L. M., J. P., E. F. P., J. E., and J. H. J.). Nine of 11 patients described a reduction in their fecal excretions leading to a reduction in their frequency of defecation or emptying of their stoma bags (all, except patients O. B., and F. V. L.). Over the two years of treatment 5 of the 7 HPN patients (Patients H. R. M., J. P., E. F. P., J. H. J., and U. D. J.) experienced 16 episodes of catheter-related bacteremia. Seven of these episodes were seen in the patient E. F. P., who had an escalating abuse of analgesics, and who was excluded from the study due to the failure to document compliance.

### 5.3. Physical Exams

The most consistent finding during physical exams in relation to GLP-2 treatment was enlargement of the stoma nipple seen in all the 9 patients with a stoma. In general, the patients detected the enlargement within a week after initiation of GLP-2 treatment, and it persisted throughout the treatment periods. The enlargement reversed toward normal size within a week after discontinuation of GLP-2 treatment.

### 5.4. Injection Site Examinations

There were no generalized skin reactions in relation to GLP-2 treatment, and in only two cases, local injection site reactions appearing as small hematomas were seen at admissions in relation to the GLP-2 injections (Patients H. R. M., and J. H. J.).

### 5.5. Standard Laboratory Results

No significant changes were seen regarding the following standard laboratory results in relation to GLP-2 treatment: hemoglobin, erythrocyte volume fraction, thrombocytes, leucocytes, sedimentation, albumin, protein, C-reactive protein, alanin-aminotransferases, alkaline phosphatases, amylase, bilirubin, urea, sodium, potassium, total or ionized calcium, and magnesium. Plasma creatinine decreased at all timepoints in relation to GLP-2 treatment (range 0.086 ± 0.024 mmol/L, Y2-W52, to 0.101 ± 0.029 mmol/L, Y2-W26) compared to baseline values (0.103 ± 0.019 mmol/L, *P* < .05). Plasma-CO_2_-total increased from 21.9 ± 3.0 mmol/L at baseline (normal range 25.0 − 32.0 mmol/L) to mean values ranging from 23.4 ± 2.4 mmol/L (Y1-W13) to 27.3 ± 4.0 mmol/L (Y2-W52) in relation to GLP-2 treatment (*P* < .001). Plasma phosphate increased from a baseline value of 0.93 ± 0.16 mmol/L (normal range 0.80 − 1.50 mmol/L) to 1.18 ± 0.23 mmol/L (Y1-W13, *P* < .05) and to 1.12 ± 0.26 mmol/L (Y1-W26, *P* < .05) during the first year of GLP-2 treatment, and increases were also seen the second year (Y2-W26: 1.13 ± 0.15 mmol/L, *P* < .05 and Y2-W52: 1.06 ± 0.211.06 ± 0.21 mmol/L, *P* < .05). The physiological basis and implications of these findings are discussed separately.

### 5.6. Quality of Life and Treatment Satisfaction

The effect of GLP-2 on quality of life evaluated by SIP is presented in Table 2. The overall SIP scores were significantly better at treatment weeks 26 and 52 during both years of treatments compared to baseline. Neither the physical or psychosocial dimensions or the independent categories gave statistically clear pictures of what caused this positive result, but in general numerical improvements were seen within all dimensions and categories at treatment weeks 26 and 52 during both years of treatments compared to baseline (Table 2). The VAS scores improved significantly at all admissions in relation to GLP-2 treatment from a baseline value of 4.1 ± 2.8 cm to values ranging from 5.1 ± 2.8 cm (Y1-W13) to 6.0 ± 2.4 cm (Y2-W52).

The effect of GLP-2 on quality of life evaluated by SF-36 is presented in Table 3. No changes were seen regarding physical function, limitation based on physical function, physical pain or general well-being, whereby no significant changes were seen in the physical component summary. No changes were seen regarding energy, social function, or limitations based on mental function in relation to GLP-2 treatment. Mental function and well-being was significantly better at weeks 13 and 52 of the first year and during weeks 13, 26, and 52 of the second year of GLP-2 treatment compared to baseline (*P* < .05). The mental component summary was significantly improved at week 52 of the first year of GLP-2 treatment compared to baseline (*P* < .05).

Numerically, the overall IBDQ scores were significantly better at all treatment points during the two years of GLP-2 treatment (*P* = .007), but the post hoc pair-wise test of differences between admission periods and the baseline period were negative (Table 4). The overall positive effects on IBDQ scores seemed to be mediated through positive effects of GLP-2 on systemic symptoms and social function, whereas bowel symptoms and emotional function was unaffected by GLP-2 treatment.

The results of the treatment satisfaction questionnaire are given in Table 5. Overall, the satisfaction with GLP-2 treatment was high, and all patients would recommend GLP-2 patients to others, who shared their symptoms. All eight patients receiving Cholylsarcosine responded that they preferred taking GLP-2 injections TID compared to consuming Cholylsarcosine capsules (2 g/day, eight capsules) TID on the assumption, that the effect was the same.

## 6. Discussion

Patients with intestinal failure frequently require life-long parenteral nutrition. Although providing good nutritional recovery, the complex technology of home parenteral nutrition may reduce the quality of life [9], and cause serious complications, such as sepsis, venous thrombosis, metabolic bone disease, renal impairment, and liver disease [10–13]. Small bowel transplantation is an exciting alternative, but it is still mainly considered for patients failing on HPN [11, 14].

It has been speculated that GLP-2 could enhance small bowel adaptation and absorption, thereby possibly increasing the quality of life by reducing fecal excretions, the need for parenteral nutrition, and the incidence of HPN-related complications. Preliminary results from “proof of concept,” short-term studies have demonstrated clinically meaningful increases in intestinal absorption in relation to 35 days of treatment with native GLP-2 [3] and 21 days of treatment with the analog teduglutide [4]. However, the effects of these treatments subsided when discontinued, and therefore long-term, possibly life-long treatment is required. In contrast to the previous short-term studies, where active efforts were made to enforce strict compliance to injections and adhesion to fixed diets in hospital-like settings, this study aimed at describing the consequences of introducing and expanding long-term GLP-2 treatment to the daily life of the short bowel patients. The open-label, uncontrolled study-design could be criticized, but since tolerability and safety were our main concern, and since GLP-2 treatment has never exceeded 35 days in humans, this setup was believed to provide valuable information prior to designing and conducting larger blinded, randomized, placebo-controlled studies.

The physiological effects of long-term GLP-2 treatment in the patients included in this study are presented separately. However, in summary, the GLP-2 treatment reduced fecal wet weight losses with around 1000 mL/day, whereby the patients needed to drink less, since the parenteral support was kept constant. The effects on energy and macronutrient absorption were minor. This enabled the patients to maintain their intestinal fluid and electrolyte absorption at lower oral intakes. A reduction in the amplitude of the daily fluctuations in the fluid-balance in relation to GLP-2 treatment may explain the beneficial effects on renal function.

GLP-2 treatment was safe and well tolerated as demonstrated by the high compliance in the SBS patients completing this study (≥94%) and also in the patients discontinuing GLP-2 treatment (≥93%). 

Considering the safety, one of the patients with Crohn's disease required elective surgery due to the evolvement of a fibrostenotic lesion at the neoterminal anastomosis. It is uncertain, whether it was induced by the GLP-2 treatment or simply reflected the natural history of the Crohn's disease in this patient. Nevertheless, it seems that caution should be taken, when prescribing GLP-2 or analogs to patients with a relative intestinal stenosis, a narrow stoma, or a history of abdominal pain. GLP-2 treatment could cause a manifest condition of bowel obstruction. In the patient O. B., aggravation of chronic abdominal pain lead to discontinuation of GLP-2 treatment. In this respect, the reversibility of the treatment effect and the short half-life of GLP-2 are appreciated. However, all patients should be informed, that mild, but normally transient, abdominal tenderness is likely to occur when introducing GLP-2 treatment. Patients with an ostomy should also be advised to enlarge the access connection to their ostomy bags, since the stoma nipple will enlarge in relation to GLP-2 treatment. The cause of this enlargement is unknown, but it could relate to increased intestinal blood flow and blood congestion in the stoma nipple [15], whereas structural and morphological changes in the gut mucosa, as described separately, are minor. 

The occurrence of two serious adverse events in relation to study-related biopsy procedures highlights that caution should be taken when obtaining biopsies from the small bowel through ostomies and questions their necessity in the presence of better non-invasive clinical endpoints. 

The general reports in the safety diaries of reductions in fecal output and increases in the need to urinate simply reflect the positive effects of GLP-2 on intestinal fluid absorption. These positive effects may, if the parenteral fluid support or the oral intake is not reduced accordingly, lead to peripheral edema (as evidenced in patients H. R. M., and E. F. P.) and theoretically to cardiac insufficiency and failure in susceptible patients. 

A high incidence of catheter-related bacteremia was recorded during GLP-2 treatment (16 episodes during two years in the 7 patients receiving H. P. N.). However, excluding the seven episodes in patient E. F. P., who could no longer manage either study medication or HPN independently, the incidence approached what is described in the Danish HPN cohort [16]. 

Surveillance of standard laboratory tests obtained during the two years of GLP-2 treatment did not raise concerns. As described separately, the reduction in plasma creatinine and the increase in plasma-CO_2_-total in relation to GLP-2 treatment probably reflect the beneficial effects of GLP-2 on renal function mediated trough a reduction in the amplitude of the daily fluctuations in the fluid-balance in relation to GLP-2 treatment. 

To describe changes in quality in life in relation to GLP-2 treatment, we used the validated global, SIP and SF-36 questionnaires, and a more disease-specific, IBDQ, questionnaire. However, not even the IBDQ questionnaire is designed to focus on specific disabilities in relation to living with a stoma and the need for parenteral support. Furthermore, a uniform response to GLP-2 treatment is unlikely to occur. In some patients, the physiological benefits of GLP-2 on intestinal function may improve their social life, whereas others may focus on the effects on physical function. In general, GLP-2 improved the overall SIP scores and VAS-scores, and numerical, but nonsignificant, improvements were also detected regarding the SF-36 and IBDQ scores. It seems that the effects of GLP-2 are most pronounced in the psychosocial and mental domains, whereas the effects on bowel symptoms and general physical functions are less pronounced. The high compliance and treatment satisfaction (Table 5) suggests that the physiological effects and the magnitudes of these changes in measures of quality of life in relation to GLP-2 treatment are clinically relevant. Currently, the annual cost of GLP-2 treatment in the doses given in this study approximates 50.000 $. Although more time consuming, qualitative interviews may be more suitable in exploring the more detailed effects of GLP-2 in the individual patients, and validated questionnaires focusing on issues more relevant for the quality of life in HPN patients are currently developed and tested. 

The patients preferred to take GLP-2 injections TID compared to consuming Cholylsarcosine capsules TID. According to all patients, long-term acceptance of 8 capsules TID would be difficult, and the clinical applicability of long-term oral Cholylsarcosine treatment in the present form and dose therefore seems limited. 

In conclusion, the main effect of GLP-2 treatment in patients with short bowel syndrome is a reduction in fecal losses of fluid and electrolytes, whereas effects on energy and macronutrient absorption are minor. This enables the patients to maintain their intestinal fluid and electrolyte absorption at lower oral intakes. Although side-effects, that is, abdominal pain and intestinal stenosis, lead to discontinuation of treatment in two patients in this study, the high compliance, treatment satisfaction, and positive trends in measures of quality of life over two years indicate that the reduction in fecal wet weight excretion of around 1000 g per day and a reduction in the need to compensate by hyperphagia/hyperdipsia in the same magnitude is desirable from a patients perspective and outdo the discomforts of injections. An alternative strategy for the HPN patients is maintaining hyperphagia/hyperdipsia in order to reduce their need for parenteral support. In this respect, the patients near both sides of the limit between intestinal insufficiency and intestinal failure are likely to achieve the largest improvements in quality of life, since treatment will either get them off or keep them off parenteral support. Based on evaluations of the HPN volumes needed in the Danish HPN-cohort, theoretically approximately 10–15% of short bowel patients with intestinal failure may be able to regain intestinal autonomy, may be weaned from H. P. N., and may have their central line removed in relation to treatment with GLP-2, 400 ug TID, provided that they are able to maintain their hyperphagia. According to most patients in this study, the benefits of GLP-2 are welcome in the limited treatments armamentarium of the short bowel syndrome. 

## Figures and Tables

**Table 1:** Patient characteristics at baseline.

Patient ID	Gender/Age (years)/Diagnosis	Body mass index (kg/m^2^)	Small bowel (cm)	Colon-in-cont. (%)	Time since last surgery (years)	Wet weight intake (kg/d)	Fecal wet weight excretion (kg/d)	Parenteral fluid (L/d)	Diet energy intake (MJ/d)	Fecal energy excretion (MJ/d)	Patenteral energy (MJ/d)	Duration of HPN (years)	Time on GLP-2 (days of 365 + 365)
HRM	F/47/CD	21.3	150	0	2.5	5.2	3.7	1.0	13.5	7.1	0.8	2.5	365 + 365
LM	M/27/CD	21.0	70	75	2.5	3.9	2.3	2.3	11.8	6.0	6.4	2.5	365 + 365
OB	M/49/CD	25.5	150	0	11.6	3.1	1.3	0.5	13.2	4.6	0.0	11.6	232 + 0, Abd. Pain
GL	M/53/CD	17.3	180	0	20.6	3.2	1.6	no	16.8	4.4	no	no	365 + 365
JP	M/24/CD	20.4	130	100	2.3	3.1	0.7	1.8	16.4	4.6	4.5	2.3	365 + 160, Abd. Pain
EFP	F/55/CD	17.5	50	0	0.5	2.2	4.9	3.1	8.0	7.1	5.5	1.7	365 + 174, unrel. Feedback
JE	M/44/UC compl.	27.2	200	0	1.6	5.9	3.9	no	13.8	5.5	no	no	365 + 365
JHJ	M/55/UC compl.	22.2	200	0	2.3	4.6	1.9	1.3	17.8	2.8	0.4	2.3	365 + 365
UDJ	F/50/UC compl	25.8	150	0	0.9	2.8	2.2	2.9	8.8	2.2	2.7	2.9	365 + 365
JV	M/67/CD	22.6	180	0	12.1	8.7	7.1	no	28.2	18.1	no	no	365 + 365
FVL	M/59/CD	20.1	290	0	5.8	3.1	1.2	no	7.8	1.3	no	no	365 + 365

M~male, F~female, CD~Crohns disease, UC compl. ~Ulcerative colitis complications, Abd. Pain ~Abdominal pain, Unrel. Feedback ~Unreliable feedback.

**Table 2:** Sickness impact profile, SIP (0% ~Best).

(mean ± SD, *n* = 8)	1st year				2nd year				
	Baseline	Week 13	Week 26	Week 52	Washout	Week 13	Week 26	Week 52	*P*-value

Treatment	None	GLP-2	GLP-2	GLP-2	None	GLP-2	GLP-2 + Cholyl-sarcosine	GLP-2	—
Overall	10.3 ± 8.9	9.7 ± 10.2	7.6 ± 11.4§	6.2 ± 9.5§§§	7.8 ± 8.3	8.6 ± 9.6	7.2 ± 10.5§	6.7 ± 8.4§§	—
Body care and movement	2.8 ± 4.1	1.0 ± 1.9	2.1 ± 3.3	1.5 ± 2.1	2.8 ± 4.1	2.6 ± 4.6	4.3 ± 5.7	2.1 ± 3.3	—
Mobility	13.8 ± 18.5	13.8 ± 20.7	7.5 ± 17.5	7.5 ± 13.9	7.5 ± 10.4	11.3 ± 18.9	12.5 ± 25.5	8.8 ± 18.1	—
Ambulation	5.3 ± 7.8	6.3 ± 9.8	1.0 ± 2.8	2.1 ± 6.0	5.3 ± 7.8	4.3 ± 7.9	3.1 ± 6.3	4.1 ± 8.9	—
Physical Dimension	5.9 ± 7.8	5.4 ± 7.6	3.0 ± 6.1	3.0 ± 5.5	4.3 ± 5.1	5.0 ± 7.6	5.8 ± 9.6	4.3 ± 7.3	—
Emotional behaviour	9.7 ± 9.3	8.3 ± 11.5	6.9 ± 15.6	4.2 ± 8.3	2.8 ± 5.1	8.3 ± 11.5	5.6 ± 11.9	6.9 ± 13.2	—
Social interaction	16.3 ± 18.7	18.1 ± 21.5	11.9 ± 18.5	8.8 ± 18.9	10.0 ± 13.6	10.0 ± 14.8	7.5 ± 12.2§	9.4 ± 10.2	—
Alertness behaviour	16.2 ± 29.2	22.5 ± 27.1	16.3 ± 21.3	13.8 ± 20.0	17.5 ± 24.3	27.5 ± 38.5	10.0 ± 16.0	16.3 ± 27.7	—
Communication	0.0 ± 0.0	0.0 ± 0.0	1.4 ± 3.9	1.4 ± 3.9	0.0 ± 0.0	0.0 ± 0.0	0.0 ± 0.0	0.0 ± 0.0	—
Psychosocial Dimension	11.9 ± 11.6	13.9 ± 15.8	9.8 ± 15.1	7.5 ± 13.7	8.3 ± 11.0	11.5 ± 11.1	6.1 ± 9.5§§	8.6 ± 11.2	—
Sleep and rest	21.4 ± 20.2	14.3 ± 13.2	23.2 ± 32.3	16.1 ± 19.4	23.2 ± 24.1	10.7 ± 14.8	21.4 ± 35.0	12.5 ± 16.1	—
Home management	11.3 ± 13.6	11.3 ± 16.4	6.3 ± 14.1	6.3 ± 10.6	8.8 ± 11.3	7.5 ± 11.9	8.8 ± 17.3	5.0 ± 10.7	—
Work	6.9 ± 13.2	0.0 ± 0.0	5.6 ± 15.7	0.0 ± 0.0	2.8 ± 7.8	6.9 ± 13.2	2.8 ± 5.1	2.8 ± 7.8	—
Recreation and pastimes	25.1 ± 29.2	25.2 ± 24.2	15.8 ± 27.4	17.4 ± 26.7	18.9 ± 26.8	20.5 ± 30.6	12.6 ± 25.9	15.8 ± 27.4	—
Eating	1.6 ± 4.4	0.0 ± 0.0	0.0 ± 0.0	1.6 ± 4.4	3.1 ± 5.8	0.0 ± 0.0	3.1 ± 5.8	1.6 ± 4.4	—
Independent Categories	12.8 ± 10.2	10.4 ± 8.7	9.3 ± 12.9	8.4 ± 9.9	10.8 ± 10.5	8.3 ± 8.6	9.8 ± 14.4	7.3 ± 8.9§	—
QOL VAS-score, 0–9 cm, 9 cm ~best	4.1 ± 2.8	5.1 ± 2.8*	5.8 ± 2.6*	5.9 ± 2.4*	4.7 ± 3.3*	5.8 ± 2.8*	5.6 ± 2.5*	6.0 ± 2.4*	0.01

QOL VAS-score ~ Quality of life Visual Analog Scale, * ~ *P* < .05, compared to baseline by Dunnett's test, § ~ *P* < .05, §§ ~ *P* < .01, §§§ ~ *P* < .001, compared to baseline by chi-square, alternatively Fisher exact test.

**Table 3:** Short form 36, SF-36 (100% ~Best).

(mean ± SD, *n* = 8)	1st year				2nd year				
	Baseline	Week 13	Week 26	Week 52	Washout	Week 13	Week 26	Week 52	*P*-value

Treatment	None	GLP-2	GLP-2	GLP-2	None	GLP-2	GLP-2 + Cholyl-sarcosine	GLP-2	
Physical Function	73 ± 23	76 ± 24	78 ± 24	77 ± 27	75 ± 24	79 ± 22	77 ± 25	78 ± 21	0.51
Limitation based on Physical Function	44 ± 42	72 ± 36	69 ± 40	72 ± 36	44 ± 42	66 ± 40	69 ± 44	63 ± 46	0.34
Physical Pain	82 ± 20	76 ± 32	81 ± 31	80 ± 25	80 ± 23	77 ± 32	79 ± 26	73 ± 37	0.19
General Well-being	51 ± 23	46 ± 20	48 ± 21	48 ± 20	47 ± 29	47 ± 22	49 ± 21	47 ± 20	0.84
*Physical Component Summary*	43 ± 11	43 ± 9	45 ± 12	43 ± 9	41 ± 10	44 ± 8	43 ± 9	44 ± 10	0.83
Energy	39 ± 31	58 ± 28	51 ± 39	55 ± 33	35 ± 31	47 ± 29	44 ± 31	51 ± 35	0.10
Social Function	83 ± 22	88 ± 27	86 ± 23	88 ± 22	80 ± 25	84 ± 26	81 ± 21	75 ± 35	0.44
Limitation based on Mental Function	71 ± 45	67 ± 40	67 ± 36	83 ± 36	63 ± 42	71 ± 42	79 ± 40	67 ± 36	0.28
Mental Function	70 ± 20	83 ± 18*	72 ± 26	80 ± 21*	70 ± 20	73 ± 23*	75 ± 22*	76 ± 20*	0.02
*Mental Component Summary*	45 ± 13	49 ± 11	48 ± 13	53 ± 11*	46 ± 12	50 ± 11	51 ± 10	46 ± 14	0.02

* ~ *P* < .05, Compared to Baseline by Dunnett's alternatively Bonferoni's test.

**Table 4:** Inflammatory Bowel Disease Questionnaire, IBDQ (7~Best).

(mean ± SD, *n* = 8)	1st year				2nd year				
	Baseline	Week 13	Week 26	Week 52	Washout	Week 13	Week 26	Week 52	*P*-value

Treatment	None	GLP-2	GLP-2	GLP-2	None	GLP-2	GLP-2 + Cholyl-sarcosine	GLP-2	
Overall	5.1 ± 0.9	5.3 ± 0.9	5.3 ± 1.0	5.4 ± 0.9	4.9 ± 1.0	5.4 ± 0.8	5.5 ± 0.8	5.4 ± 0.9	0.007
Bowel Symptoms	5.3 ± 0.6	5.5 ± 0.8	5.3 ± 0.8	5.5 ± 0.7	5.2 ± 0.8	5.5 ± 0.6	5.5 ± 0.8	5.4 ± 0.7	0.61
Systemic Symptoms	4.0 ± 1.4	4.5 ± 1.6	4.9 ± 1.4	4.7 ± 1.2	3.7 ± 1.6	4.2 ± 1.4	4.4 ± 1.3	4.9 ± 1.4	0.03
Emotional Function	5.5 ± 1.1	5.7 ± 1.1	5.6 ± 1.2	5.7 ± 1.1	5.5 ± 1.1	5.7 ± 0.8	5.9 ± 0.8	5.7 ± 1.0	0.44
Social Function	5.0 ± 1.3	5.0 ± 1.5	5.2 ± 1.4	5.1 ± 1.4	4.5 ± 1.4	5.3 ± 1.5	5.4 ± 1.1	5.3 ± 1.4	0.03

* ~ *P* < .05, compared to baseline by Dunnett's test.

**Table 5:** Evaluation of treatment satisfaction.

1~Totally Agree, 2~Agree a lot, 3~Agree, 4~Disagree, 5~Disagree a lot, 6~Totally disagree	(*n* = 8, mean ± SD)
1. GLP-2 allows me to do, what I please to do.	2.8 ± 1.7
2. I would definitely recommend the GLP-2 medication to others who share my symptoms.	1.1 ± 0.4
3. I am not satisfied with the medication I current receive for my symptoms.	5.1 ± 1.2
4. I am satisfied with the rapid onset of action of GLP-2.	1.9 ± 1.0
5. I feel, that GLP-2 is the best medication available on the market for me.	2.1 ± 1.8
6. I am satisfied with the GLP-2 medication that I have received for my symptoms.	1.5 ± 0.8
7. The medication enables med to eat and drink whatever I please.	2.4 ± 1.7

## References

[1] Byrne TA, Wilmore DW, Iyer K (2005). Growth hormone, glutamine, and an optimal diet reduces parenteral nutrition in patients with short bowel syndrome: a prospective, randomized, placebo-controlled, double-blind clinical trial. *Annals of Surgery*.

[2] Drucker DJ, DeForest L, Brubaker PL (1997). Intestinal response to growth factors administered alone or in combination with human [Gly2]glucagon-like peptide 2. *American Journal of Physiology*.

[3] Jeppesen PB, Hartmann B, Thulesen J (2001). Glucagon-like peptide 2 improves nutrient absorption and nutritional status in short-bowel patients with no colon. *Gastroenterology*.

[4] Jeppesen PB, Sanguinetti EL, Buchman A (2005). Teduglutide (ALX-0600), a dipeptidyl peptidase IV resistant qlucagon-like peptide 2 analogue, improves intestinal function in short bowel syndrome patients. *Gut*.

[5] Bergner M, Bobbitt RA (1981). The sickness impact profile: development and final revision of a health status measure. *Medical Care*.

[6] Ware JE, Sherbourne CD (1992). The MOS 36-item short-form health survey (SF-36)—I: conceptual framework and item selection. *Medical Care*.

[7] Guyatt G, Mitchell A, Irvine EJ (1989). A new measure of health status for clinical trials in inflammatory bowel disease. *Gastroenterology*.

[8] Heydorn S, Jeppesen PB, Mortensen PB (1999). Bile acid replacement therapy with cholylsarcosine for short-bowel syndrome. *Scandinavian Journal of Gastroenterology*.

[9] Jeppesen PB, Langholz E, Mortensen PB (1999). Quality of life in patients receiving home parenteral nutrition. *Gut*.

[10] O'Keefe SJD, Burnes JU, Thompson RL (1994). Recurrent sepsis in home parenteral nutrition patients: an analysis of risk factors. *Journal of Parenteral and Enteral Nutrition*.

[11] Jeppesen PB, Staun M, Mortensen PB (1998). Adult patients receiving home parenteral nutrition in Denmark from 1991 to 1996: who will benefit from intestinal transplantation?. *Scandinavian Journal of Gastroenterology*.

[12] Beers TR, Burnes J, Fleming CR (1990). Superior vena caval obstruction in patients with gut failure receiving home parenteral nutrition. *Journal of Parenteral and Enteral Nutrition*.

[13] Bowyer BA, Fleming CR, Ludwig J, Petz J, McGill DB (1985). Does long-term home parenteral nutrition in adult patients cause chronic liver disease?. *Journal of Parenteral and Enteral Nutrition*.

[14] Langnas AN (2004). Advances in small-intestine transplantation. *Transplantation*.

[15] Guan X, Stoll B, Lu X (2003). GLP-2-mediated up-regulation of intestinal blood flow and glucose uptake is nitric oxide-dependent in TPN-fed piglets. *Gastroenterology*.

[16] Ugur A, Marashdeh BHS, Gottschalck I, Mortensen PB, Staun M, Jeppesen PB (2006). Home parenteral nutrition in Denmark in the period from 1996 to 2001. *Scandinavian Journal of Gastroenterology*.

